# A Combination of *CD28* (rs1980422) and *IRF5* (rs10488631) Polymorphisms Is Associated with Seropositivity in Rheumatoid Arthritis: A Case Control Study

**DOI:** 10.1371/journal.pone.0153316

**Published:** 2016-04-19

**Authors:** Lucia Vernerova, Frantisek Spoutil, Miroslav Vlcek, Katarina Krskova, Adela Penesova, Milada Meskova, Andrea Marko, Katarina Raslova, Branislav Vohnout, Jozef Rovensky, Zdenko Killinger, Ivana Jochmanova, Ivica Lazurova, Guenter Steiner, Josef Smolen, Richard Imrich

**Affiliations:** 1 Institute of Clinical and Translational Research, Biomedical Centre, Slovak Academy of Sciences, Bratislava, Slovakia; 2 Institute of Molecular Genetics, Czech Academy of Sciences, Prague, Czech Republic; 3 Institute of Experimental Medicine, Czech Academy of Sciences, Prague, Czech Republic; 4 Slovak Medical University, Bratislava, Slovakia; 5 National Institute of Rheumatic Diseases, Piešťany, Slovakia; 6 5th Department of Internal Medicine, Medical Faculty of Comenius University, Bratislava, Slovakia; 7 1st Department of Internal Medicine, Faculty of Medicine, Pavol Jozef Šafárik University in Košice, Košice, Slovakia; 8 Department of Internal Medicine III, Division of Rheumatology, Medical University of Vienna, Vienna, Austria; Centro di Riferimento Oncologico, IRCCS National Cancer Institute, ITALY

## Abstract

**Introduction:**

The aim of the study was to analyse genetic architecture of RA by utilizing multiparametric statistical methods such as linear discriminant analysis (LDA) and redundancy analysis (RDA).

**Methods:**

A total of 1393 volunteers, 499 patients with RA and 894 healthy controls were included in the study. The presence of shared epitope (SE) in *HLA-DRB1* and 11 SNPs (*PTPN22* C/T (rs2476601), *STAT4* G/T (rs7574865), *CTLA4* A/G (rs3087243), *TRAF1/C5* A/G (rs3761847), *IRF5* T/C (rs10488631), *TNFAIP3* C/T (rs5029937), *AFF3* A/T (rs11676922), *PADI4* C/T (rs2240340), *CD28* T/C (rs1980422), *CSK* G/A (rs34933034) and *FCGR3A* A/C (rs396991), rheumatoid factor (RF), anti–citrullinated protein antibodies (ACPA) and clinical status was analysed using the LDA and RDA.

**Results:**

*HLA-DRB1*, *PTPN22*, *STAT4*, *IRF5* and *PADI4* significantly discriminated between RA patients and healthy controls in LDA. The correlation between RA diagnosis and the explanatory variables in the model was 0.328 (Trace = 0.107; F = 13.715; P = 0.0002). The risk variants of *IRF5* and *CD28* genes were found to be common determinants for seropositivity in RDA, while positivity of RF alone was associated with the *CTLA4* risk variant in heterozygous form. The correlation between serologic status and genetic determinants on the 1st ordinal axis was 0.468, and 0.145 on the 2nd one (Trace = 0.179; F = 6.135; P = 0.001). The risk alleles in *AFF3* gene together with the presence of ACPA were associated with higher clinical severity of RA.

**Conclusions:**

The association among multiple risk variants related to T cell receptor signalling with seropositivity may play an important role in distinct clinical phenotypes of RA. Our study demonstrates that multiparametric analyses represent a powerful tool for investigation of mutual relationships of potential risk factors in complex diseases such as RA.

## Introduction

Genetic factors have a substantial role in development of rheumatoid arthritis [RA] accounting for 50–60% of disease susceptibility [1]. For the past four decades, the strongest genetic association with RA has been attributed to human leukocyte antigen (HLA) region at chromosome 6p21, particularly to *HLA-DRB1* locus [2]. Recently, 101 non-HLA loci have been confirmed in trans-ethnic meta-analysis of RA [3]. In the population-specific genetic risk model, the 100 RA risk loci outside of the major histocompatibility complex (MHC) region [4] explained 5.5% and 4.7% of heritability in Europeans and Asians, respectively. Lately, RA has been divided into two clinical phenotypes based on the presence or absence of rheumatoid factor (RF) and antibodies against citrullinated proteins (ACPA) [5, 6]. These two clinical subtypes appear to have distinct genetic aetiologies [7]. Significant differences have been found in frequency of risk alleles in the HLA region and in *PTPN22*, *CCR6*, *CD40*, *RASGRP1* and *TAGAP* genes between ACPA-positive and ACPA-negative RA patients [8, 9].

Traditionally, genetic markers have been considered independent risk factors in majority of studies in RA. Although, this univariate approach has been successful in identifying alleles with relatively strong associations with the disease or its subtypes, interactions occurring in complex biological systems can be overlooked [10]. It remains unclear whether or not a combination of known genetic loci confers higher risk for RA development, clinical outcome or response to therapy compared to their simple additive effects. To solve this kind of question, multiparametric approaches may represent a potential tool enabling analysis of complex relationships such as those in the multifactor RA pathogenesis. The multiparametric methods have been used mainly in studies investigating predictive genetic tests in RA. In a pioneering study, McClure and colleagues found that a combination of five confirmed risk loci significantly increased an association with RA compared to the presence of any risk allele alone [11]. Subsequently, several other reports outlined predictive models for RA using HLA alleles, SNPs and clinical factors generating an aggregate weighted genetic risk score formed from the product of individual-locus odds ratios (ORs) [12, 13]. Recently, validated environmental factors such as tobacco smoking and gene-environment interactions were added to the RA risk modelling [14, 15]. These studies demonstrate that combining risk factors has a potential to provide a clinically relevant prediction with respect to disease onset [15].

The receiver operating characteristic curve analysis was adopted in studies to evaluate the performance of predictive genetic testing [16]. Various other methods have been used to combine multiple predictors for the ROC curve analysis. Among these, the most commonly used have been the allele counting methods and logistic regression [17, 18]. In order to elucidate the genetic architecture of RA, the main goal of our study was to study interactions of known genetic risk factors with serologic and clinical parameters by utilizing multiparametric statistical methods: the multivariate linear discriminant analysis (LDA) and the redundancy analysis (RDA). These multivariate ordination analyses have been already used in genome-wide association studies for correction of population stratification [19, 20]. The LDA and, in particular RDA, enables to quantify dependence between two groups of variables; independent and dependent (e.g. genes and clinical parameters) compared to the principal component analysis. Unlike regression methods, the LDA and RDA allow working with all variables with unknown correlations among them, reducing risk of finding false positive result based on a specific sample selection instead of on real differences in the whole population. Also, interpretation of such results is substantially improved, as not only relations between dependent and independent variables but also inside these groups are detected.

## Subjects and Methods

### Patients and Controls

A total of 1393 volunteers, 499 patients with RA and 894 healthy controls were included in the study. All RA patients fulfilled the 2010 ACR-EULAR classification criteria for RA [21]. The patients were recruited from the National Institute of Rheumatic Diseases, Piestany and from ten outpatient rheumatology clinics in Slovakia. Control subjects were recruited from collaborating institutes and 485 DNA samples of geographically- and sex-matched healthy individuals were obtained from DNA bank of the Slovak Medical University in Bratislava. All subjects gave informed written consent and the study was approved by the Ethics Committee of the National Institute of Rheumatic Diseases, Piestany, Slovakia in agreement with the ethical guidelines of the Declaration of Helsinki as revised in 2000. Disease activity score 28 (DAS28) was calculated from number of swollen and tender joints and erythrocyte sedimentation rate [22]. The health assessment questionnaire disability index (HAQ-DI) was recorded using the standardized form [23]. Rheumatoid factor (RF), anti-citrullinated peptides antibodies (ACPA), C-reactive protein (CRP) and erythrocyte sedimentation rate (ESR) were measured in accredited hospital laboratories. IgM-rheumatoid factor was measured by nephelometry (CSL Behring, Germany), IgA-RF by ELISA (Hycor, Indianapolis, IN, USA), and ACPA by ELISA (Phadia, Freiburg, Germany). RA patients were considered RF positive when IgM or IgA-RF was positive. RA patients were considered seropositive when either RF or ACPA were above a specific threshold as given by the manufacturer. Clinical characteristics of the cohort are shown in Table 1.

**Table 1:** Basic demographics of RA cases and healthy controls.

	Association analysis		Clinical analysis
Diagnosis	RA n = 499	HC n = 894	RA n = 220
**Age (years)**	57.3±12.1	41.2±12.1	58.1±11.5
**Smoking (%)**	13.6 (N = 308)	27.9 (N = 506)	11.8
**Disease duration (years)**	11.9±9.1 (N = 423)		12.1±8.9
**RF**^a^ **positive (%)**	72.6 (N = 351)		75
**ACPA**^b^ **positive (%)**	65.5 (N = 313)		68.6
**CRP**^c^ **(mg/l)**	11.7±15.2 (N = 398)		13.5±16.3
**DAS28**^d^			4.6±1.4
**Swollen joint count**			4.4±5.1
**Tender joint count**			8.4±7
**Morning stiffness (min)**			40.9±42.6
**Glucocorticoids treated (%)**			59.1
**Methotrexate treated (%)**			61.4
**Biological treatment (%)**			9.1
**NSAID**^e^ **treated (%)**			82.7

Data are expressed as mean ± SD.

^a^RF, Rheumatoid factor.

^b^ACPA, Anti-citrullinated peptide antibodies.

^c^CRP, C-reactive protein.

^d^DAS28, disease activity score 28.

^e^NSAID, non-steroid anti-inflammatory drugs.

### HLA Typing

DNA was isolated from peripheral blood by FlexiGene DNA Kit (Qiagen, Hilden, Germany). The detection of alleles in *HLA-DRB1* gene was performed using ELPHA HLA DRB1 DL ALL 48 system (Bio-Rad, Dreieich, Germany). For PCR amplification of genomic DNA NovaTaqTM DNA polymerase (Novagen (Merck KGaA), Darmstadt, Germany) and nucleotide mixture dNTP mix (Novagen (Merck KGaA), Darmstadt, Germany) were used apart from primers and PCR buffer included in ELPHA kit. The assay starting from hybridization step till the final fluorescence reading was performed on QuickStep ELISA Processor (Bio-Rad, Dreieich, Germany). The final combination of *HLA-DRB1* alleles in particular samples was evaluated by Bio-Rad HLA Typing Software (Bio-Rad, Dreieich, Germany). HLA-DRB1*04 alleles (*0401, *0404, *0405, *0408), HLA-DRB1*0101 and *0102, HLA-DRB1*1402 and HLA-DRB1*1001 were considered shared epitope coding alleles (SE alleles) [24].

### SNP Genotyping

SNP genotyping of 11 genes *PTPN22 C/T* (rs2476601), *STAT4 G/T* (rs7574865), *CTLA4 A/G* (rs3087243), *TRAF1/C5 A/G* (rs3761847), *IRF5 T/C* (rs10488631), *TNFAIP3 C/T* (rs5029937), *AFF3 A/T* (rs11676922), *PADI4 C/T* (rs2240340), *CD28 T/C* (rs1980422), *CSK G/A* (rs34933034) and *FCGR3A A/C* (rs396991) was performed using TaqMan^®^ SNP Genotyping Assays (Applied Biosystems) and TaqMan^®^ Genotyping PCR Master Mix (Applied Biosystems) on ABI 7900HT Fast Real-Time PCR System (Applied Biosystems). The results were analysed by Primer Express^™^ software (Applied Biosystems).

### Statistical Analysis

Statistical analysis of obtained data was performed using SPSS v11.5 software (SPSS Inc., Chicago, IL, USA). All SNPs were individually tested for association with RA by Chi square test (χ^2^). Odds ratio with 95% confidence interval was calculated using logistic regression. Multivariable ordination analysis was performed in Canoco 4.5 software (Microcomputer Power, NY, USA). The categorical data were coded as dummy-variables (i.e. each category as new variable with 0/1 values). All variables were Z-normalized. As the analysis cannot operate with blank fields in the table, those variables and subjects with the most missing values had to be excluded from the analysis. Before the main analysis, the detrended correspondence analysis (DCA) or detrended correspondence canonical analysis (DCCA) were performed to assure that PCA, LDA or RDA could be applied according to the 1^st^ ordinal axis gradient length. PCA was performed for each group of variables to see relations among variables in the group. Monte-Carlo permutation test with 999 permutations was applied as a method of forward selection of significant dependent variables in LDA and RDA. This approach helped us to avoid a problem with possible correlations among numerous independent variables (e.g. genes), as the best fitting variable was chosen by the method as the first; and the second (and further) variables were selected only if their presence improved the model. In the final model, the variables with significant influence were supplemented with variables correlated with them (including remaining stages of the variable). These ones do not explain any new variability of the model, so their influence is negligible, but they can help us to compare our results to previously published observations. First, LDA was performed to see, which genetic factors separate patients with RA from healthy controls. Second, RDA was performed under similar conditions, however, healthy controls and RA patients were split up into two groups according to the presence of antibodies. Third, another RDA was performed with genetic factors and smoking as independent variables, and with clinical parameters associated with RA diagnosis as dependent ones. Age and sex were included as covariates.

## Results

### Analysis of Genetic Factors Associated with RA

**Fig 1:**
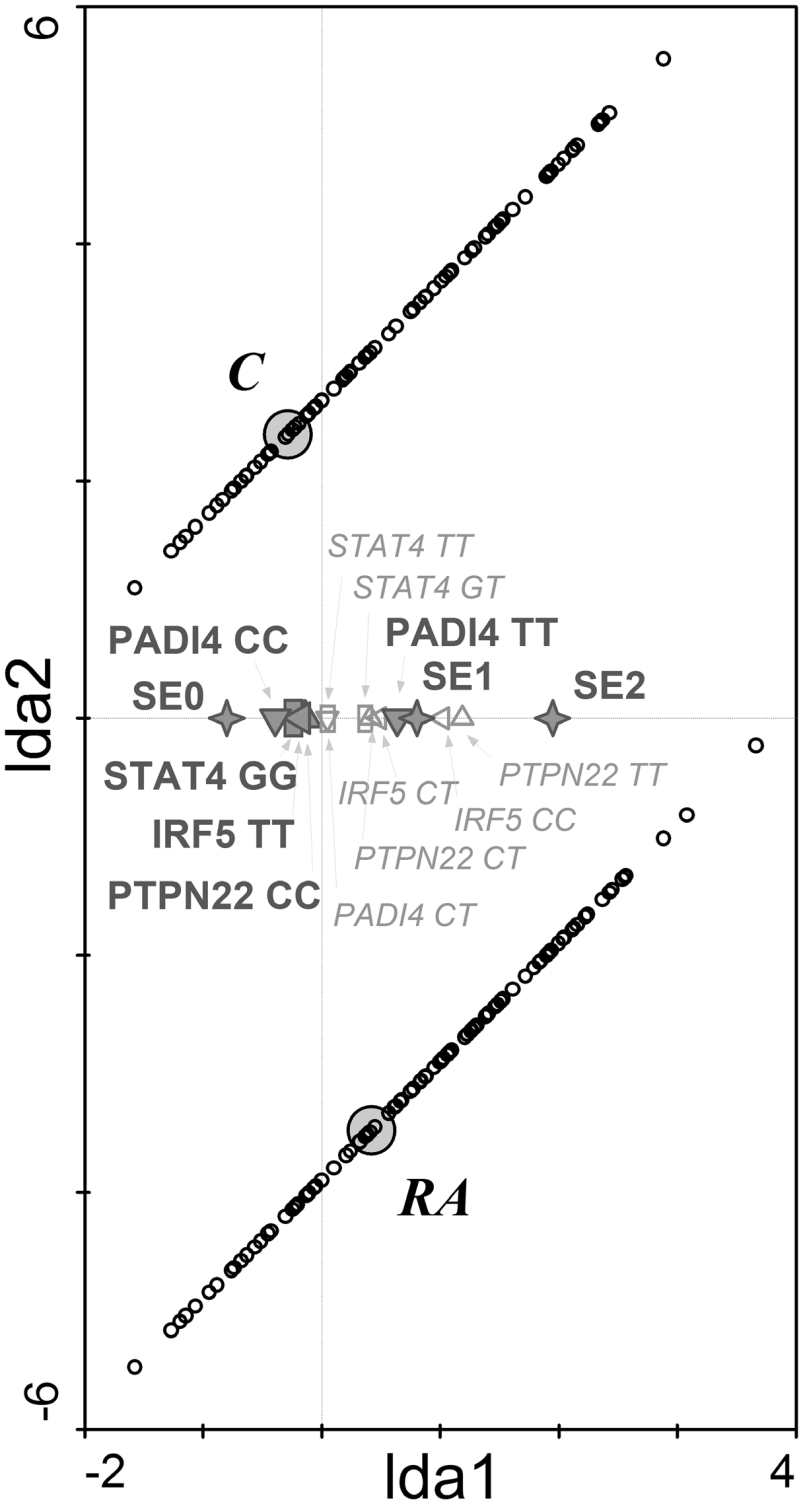
The genetic discrimination of RA patients and controls. Linear discrimination analysis diagram shows that shared epitope and single nucleotide polymorphisms in PTPN22, STAT4, IRF5 and PADI4 genes significantly discriminated between RA patients and healthy controls. RA—RA patients; C—control group; SE (0,1,2)—number of SE coding allele in HLA-DRB1 gene (✧); IRF5 (CC, CT, TT)—genotypes in IRF5 gene (C risk allele) (◁); PADI4 (TT, CT, CC)–genotypes in PADI4 gene (T risk allele) (▽); PTPN22 (CC, CT, TT)–genotypes in PTPN22 gene (A risk allele) (△); STAT4 (GG, GT, TT)–genotypes in STAT4 gene (T risk allele) (☐). Diagram reading clue: Small circles represent individual cases. Large grey circles—centroids—represent subject groups (RA patients and controls). Symbols are genetic factors. Large bold symbols represent genotypes significantly influencing the distribution of subjects. Small empty symbols represent other genotypes of selected genes. The closer to the group centroid the gene symbol lies, the stronger is its impact on the classification of subjects to particular group.

All genes analysed in the study were in Hardy-Weinberg equilibrium. The presence of SE coding alleles in *HLA-DRB1* gene was significantly higher in RA patients compared to controls (Table 2). The most frequent SE alleles in patient group were DRB1*01:*01* (13.4%), DRB1*04:*01* (11.9%), DRB1*04:*04* (3.8%) (data not shown). Among 11 studied SNPs, risk allele and genotype frequencies of *PTPN22*, *STAT4*, *IRF5* and *PADI4* genes were significantly higher in RA patients compared to controls (Table 2). LDA model was used to classify our cohort based on the genotype frequencies of studied SNPs. A major discriminant function was generated based on the variances seen in the data using LDA. This discriminant function was assigned to 1st ordinal axis of LDA diagram and the centroids for diagnosis were plotted in this matrix together with the significant genetic determinants (Fig 1). Based on these discriminant functions, the model predicted to which of the study group individual subject belongs with an overall accuracy of 10.7%. The correlation coefficient between RA diagnosis and explanatory variables in the model was 0.328 (Trace = 0.107; F = 13.715; P = 0.0002). These genetic determinants were selected as significant in the following order representing their importance in the model: SE 0 > *IRF5* TT > SE 1 + SE 2 > *STAT4* GG *> PADI4* CC *+ PADI4* TT > *PTPN22* CC.

**Table 2:** Allelic frequencies of all studied genes and their association with RA.

SNP^a^		Allele		Allele frequency (%)		Association with RA (χ^2^^c^)	
Gene	ID^b^	Major	Minor	RA	C	p value	OR^d^ (95% CI^e^)
***HLA-DRB1***	-	-	-	-	-	<0.0001	2.54 (2.1–3.08)
***PTPN22***	rs2476601	C	T	14.6	10.8	0.003	1.43 (1.13–1.80)
***STAT4***	rs7574865	G	T	26.8	22.0	0.004	1.30 (1.09–1.56)
***CTLA4***	rs3087243	A	G	38.3	40.5	0.253	1.10 (0.93–1.30)
**TRAF1/C5**	rs3761847	A	G	41.4	43.4	0.308	0.92 (0.79–1.08)
**TNFAIP3**	rs5029937	C	T	2.2	2.5	0.599	0.87 (0.52–1.47)
***AFF3***	rs11676922	A	T	46.9	47.2	0.889	0.99 (0.84–1.16)
***IRF5***	rs10488631	T	C	15.4	11.6	0.004	1.41 (1.12–1.78)
***PADI4***	rs2240340	C	T	45.9	38.9	0.001	1.32 (1.13–1.55)
***CD28***	rs1980422	T	C	27.2	25.6	0.382	1.08 (0.90–1.30)
***CSK***	rs34933034	G	A	17.8	19.3	0.377	0.91 (0.74–1.12)
**FCGR3A**	rs396991	A	C	38.1	37.2	0.637	1.04 (0.88–1.23)

^a^SNP, single nucleotide polymorphism.

^b^ID, identification number.

^c^χ^2^, Chi-square test.

^d^OR, Odds ratio.

^e^95% CI, 95% confidence interval.

In total, the presence of RA significantly correlated with genetic determinants *PADI4* and SE alleles. As shown in Fig 1, at least one SE allele and two risk alleles (T) in *PADI4* gene were the most important combination associated with RA diagnosis in our cohort. There was a strong prevalence of the non-risk genotype in genes *PADI4*, *STAT4*, *IRF5* and *PTPN22* and lack of SE coding alleles in control groups.

### Analysis of Genetic Factors Associated with Autoantibody Status in RA

An association analysis between antibody status and genetic markers was performed in RA patients. As expected, seropositivity was associated with SE alleles [OR (95% CI): 1.75 (1.13–2.24) p<0.0001] and presence of risk genotype in *IRF5* [OR (95% CI): 1.54 (1.07–2.23); p = 0.021]. RF positivity was significantly associated with SE alleles [OR (95% CI): 2.43 (1.62–3.64); p<0.0001] and the presence of risk genotype in *IRF5* gene [OR (95% CI): 1.76 (1.06–2.95); p = 0.030] was observed. Similarly, ACPA positivity was associated with SE alleles [OR (95% CI): 3.25 (2.14–4.95); p<0.0001] and with risk genotype in *IRF5* [OR (95% CI): 2.28 (1.35–3.85); p = 0.002], and a trend was observed also in *CD28* gene [OR (95% CI): 1.45 (0.98–2.16) p = 0.064].

**Fig 2:**
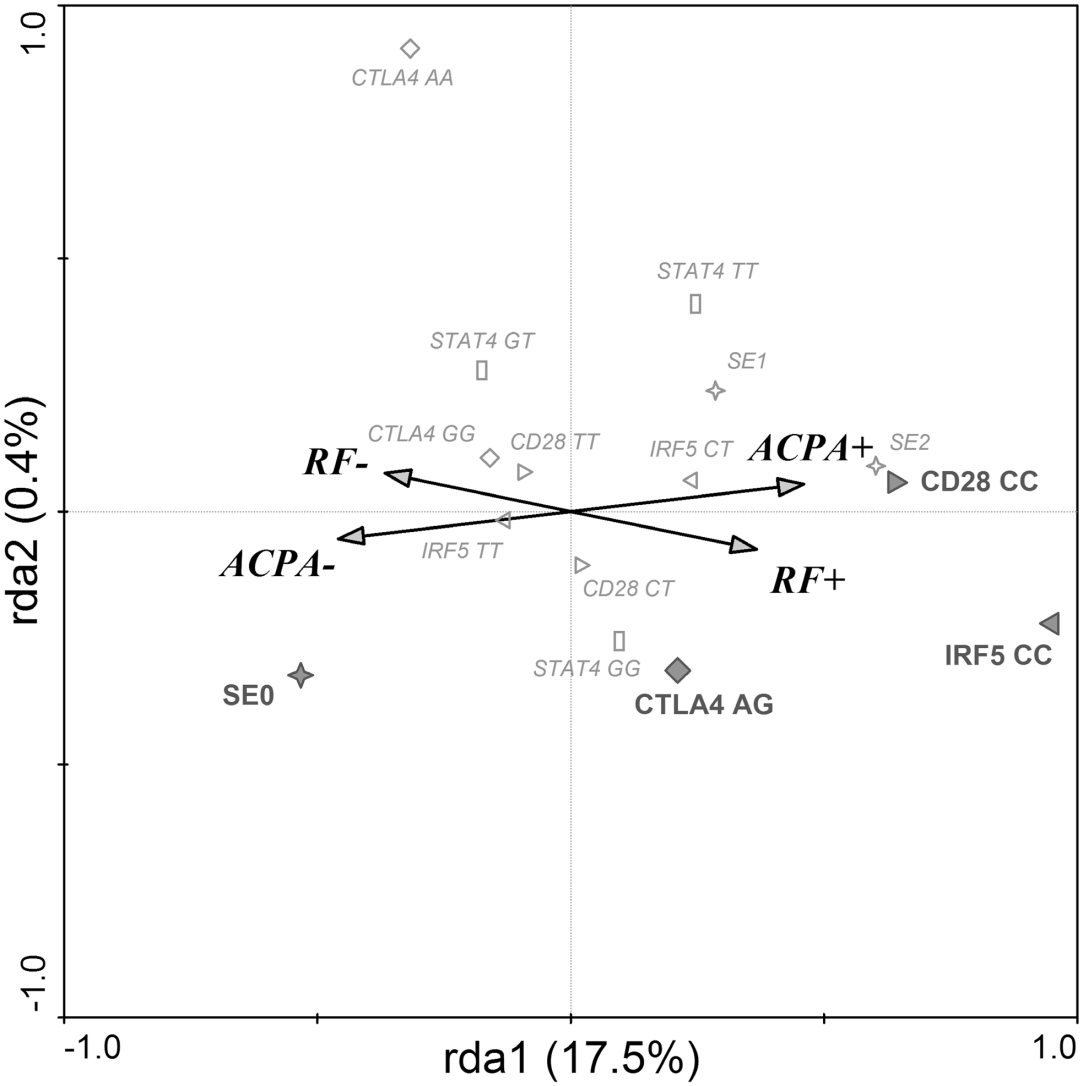
SNPs associated with seropositivity in RA. Redundancy discrimination analysis plot showing that IRF5, CD28 and CTLA4 are associated with seropositivity in RA patients. RF+–rheumatoid factor positive RA patients; RF-–rheumatoid factor negative RA patients; ACPA+–anti-citrullinated peptides antibodies positive RA patients; ACPA-–anti-citrullinated peptides antibodies negative RA patients; SE (0,1,2)—number of shared epitope coding alleles in HLA-DRB1 gene (✧); IRF5 (CC, CT, TT)—genotypes in IRF5 gene (C risk allele) (▷); CD28 (CC, CT, TT)–genotypes in CD28 gene (C risk allele) (◁); CTLA4 (AG, GG, AA)–genotypes in CTLA4 gene (G risk allele) (◊). Diagram reading clue: Symbols are genetic factors. Large bold symbols represent genotypes significantly influencing the presence of RF and ACPA. Small empty symbols represent other genotypes of selected genes. Direction of arrow indicates which serologic status is associated with the genetic parameters and the length of the arrow indicates the magnitude of the association.

The RDA, which estimated the effect of SNPs on the presence of antibodies, found a significant model explaining 17.9% of variability in data (1st ordinal axis 17.5% and 2nd ordinal axis 0.4%) (Fig 2). The correlation between categories RF status, ACPA status and genetic determinants on the 1st ordinal axis is 0.468, and on the 2nd ordinal axis 0.145 (Trace = 0.179; F = 6.135; P = 0.001). RF and ACPA negative RA patients were more likely to have SE 0 genotype. On the other hand, *CD28* CC and *IRF5* CC genotypes were associated with both RF and ACPA positivity, while *CTLA4* AG genotype associated preferentially with RF positivity.

### Analysis of Genetic Factors Associated with Clinical Outcomes

**Fig 3:**
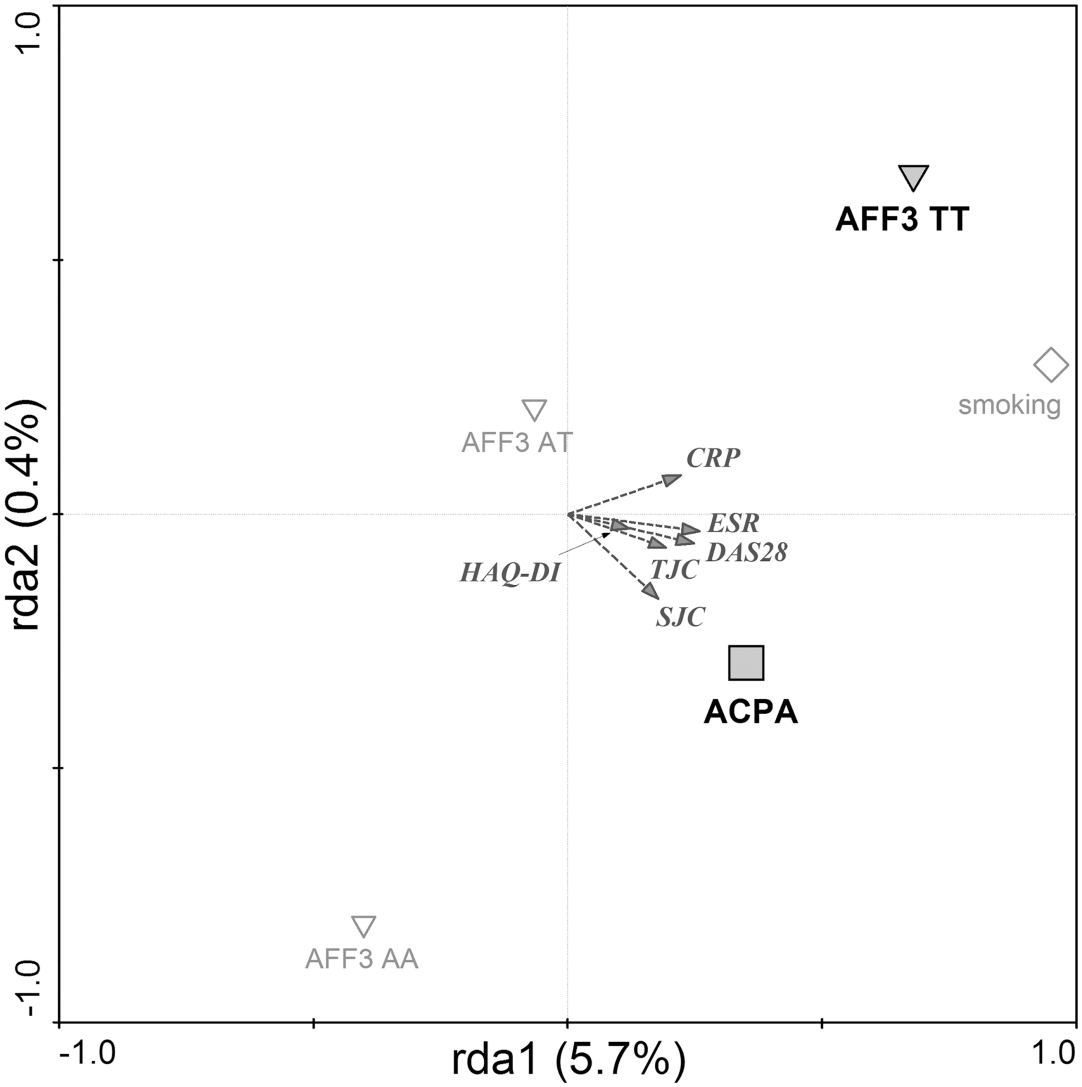
Factors associated with clinical severity in RA. Redundancy analysis plot showing that risk alleles in AFF3 gene, together with ACPA positivity are associated with higher clinical severity of RA. ACPA—anti-citrullinated peptides antibodies (□); *AFF3* (TT, AT, AA)–genotypes in *AFF3* gene (T risk allele) (▽). Diagram reading clue: Symbols are genetic and serologic factors. Large bold symbols represent genotypes and antibody presence significantly influencing the clinical parameters of disease severity (DAS28, CRP, ESR, TJC, SJC, HAQ-DI). Small empty symbols represent other factors and genotypes of selected genes. Direction of arrow indicates which of the clinical factors are associated with the genetic and serologic parameters and the length of the arrow indicates the magnitude of the association.

In general, RDA analysis with forward selection performed in RA patients showed that ACPA positivity, *AFF3* gene, in particular *AFF3* TT genotype and smoking significantly associate with higher disease activity defined by clinical parameters DAS28, CRP, ESR, TJC, SJC and HAQ-DI. The explanatory variables explain 6.1% of variability among RA patients (Trace = 0.061; F = 3.482; P = 0.0020). In particular, higher CRP levels were associated with the presence of *AFF3* TT genotype, whereas higher DAS28 and higher number of swollen and tender joints were correlated with ACPA positivity (Fig 3).

## Discussion

The aim of the presented study was to analyse possible interactions among genetic, serologic and clinical data in a cohort of Slovak patients with RA. LDA and RDA, multiparametric statistical methods, together with conventional association analyses of known RA risk gene variants were applied. As expected, among eleven gene loci previously associated with RA, risk variants in *IRF5*, *STAT4*, *PADI4* and *PTPN22* genes and the presence of SE alleles in *HLA-DRB1* were found to be significantly more frequent in RA patients compared to controls in our study. In line with the latter, the LDA model demonstrated that at least one SE allele and two risk alleles (T) in the *PADI4* gene is the most significant determinant of RA diagnosis. On the other hand, the presence of non-risk genotypes in genes *PADI4*, *STAT4*, *IRF5* and *PTPN22* and the lack of SE coding alleles as determinants of non-RA phenotype in our study may suggest a cluster of protective gene variants preventing RA development. It remains unclear whether or not the gene cluster can be regarded RA protective in truly mechanistic way.

Our results suggest that only *HLA-DRB1* and *PADI4* are predisposing to RA, whereas the other four genotypes were dominant in the healthy population. Based on this analysis, the presence of non-risk genotypes in *IRF5*, *STAT4*, *PADI4* and *PTPN22* indicates more strongly the exclusion of RA diagnosis than would the risk genotype indicate its confirmation. The presence of at least one shared epitope coding allele in *HLA-DRB1* gene among RA predisposing factors is not surprising since the HLA region, and particularly the *HLA-DRB1* locus, is considered the strongest genetic factor contributing to the increased susceptibility of RA. *PADI4* is, as one of the isoenzymes responsible for post-translational conversion of arginine to citrulline, one of the most interesting genes associated with RA. However, its association with disease remains rather disputable. The association of *PADI4* with RA was confirmed in European as well as Japanese populations [25] showing also high specificity for the disease. On the other hand, it failed to be confirmed as RA-associated gene variant in a large British-French-Spanish cohort, calling its association in European population in question [26, 27]. Our results support the association of SNP in *PADI4* gene with RA in a population of European origin. Although there might be a correlation of *PADI4* with ACPA positivity, the presence of both components in significant LDA model in our study indicates their own contribution to data variability and thus proves them as two separate factors.

The presence or absence of seropositivity appears to be fundamental with regard to a clinical phenotype of RA [5, 6]. Therefore, we performed an analysis investigating which genes from the studied group are associated with RF and/or ACPA positivity in our RA cohort. We found a significant correlation of RA seropositivity with *IRF5* and *CD28* risk allele homozygosity. As shown on the RDA diagram [Fig 2], the risk variants of *IRF5* and *CD28* genes are common determinants for both RF- and ACPA-positivity. In other words, the presence of both risk gene variants has a significant association with seropositivity. Moreover, the RDA shows that RF-positivity is associated with the *CTLA4* risk variant in heterozygous form. The association of *IRF5* gene, including the rs10488631 SNP analysed in this study was mainly described in seronegative RA [28, 29]. In other studies, however, this SNP was associated with the disease exclusively in ACPA positive RA patients and in autoantibody positive RA replication cohort [30, 31]. Interestingly, the absence of SE alleles associated with ACPA negativity was more significant than the association of two SE alleles with seropositivity. It has been shown that SE is strongly associated with both RF status and presence of ACPA [6, 32]. More specifically, ACPA play a vital role in the causal pathway between SE and joint erosions [33].

IRF5 is a latent transcription factor expressed in lymphocytes, macrophages and dendritic cells. IRF5 is involved in expression regulation of IFN-α genes and mediates Toll-like receptor (TLR) signal transduction leading to production of proinflammatory cytokines [34]. CTLA-4 and CD28 are functionally as well as structurally related, as both bind the same counter-receptor, the two B7 family members (CD80 and CD86), that are present on antigen presenting cells, but the molecules have opposing effects on T cell activation playing an important role in the T cell receptor (TCR) signalling regulation [35]. The *CTLA4* gene was repeatedly found associated with a diagnosis of RA in genome-wide association studies [31, 36, 37], however, various SNPs in this gene were analysed. In case of CT60 SNP included in this study, the results are contradictory [38, 39]. Among these *CTLA4* SNPs, the CT60 variant appears to have the highest functional relevance; the G allele is associated with lower mRNA and protein levels of soluble *CTLA4* that could result in increased T cell activation. The allelic distribution of *CTLA4* gene was very similar in our cohort (A 61.7; G 38.3%) indicating highest prevalence of heterozygotes among genotypes (AA 38%, AG 47% and GG 15%). Lower significance of homozygotic states of *CTLA4* in the final model could be contributed to lower statistical power because of their smaller counts. Surprisingly, *CTLA4* and *CD28* genes did not show association with RA in our study when analysed as independent risk factors. The lack of independent association of AFF3, CD28, CTLA4, IRF5 and TNAIP3 genes in our cohort might be partially explained by observations of Viatte and co-authors [40], who described the association of these SNPs with RA only in ACPA positive patients. This, together with the fact that the association analysis in this study was done on the whole group of patients regardless the antibody status, with 34.5% of ACPA negative patients from 499 cases analysed, might rationalize why these genes did not reach statistical significance.

In the present study, the RDA was used to investigate a combination of factors associated with the disease activity in RA expressed by DAS28, CRP, ESR, swollen and tender joint counts and HAQ-DI. Our results demonstrate that from all factors analysed, ACPA positivity and the homozygous state of *AFF3* risk alleles most significantly influenced the disease activity in our cohort of patients. The link of serologic status to different disease outcomes, with more severe and erosive disease observed in RF and ACPA positive patients is well known [41–43]. In terms of disease activity, to our best knowledge, AFF3 locus has been previously associated with the rate of joint destruction [44] but no other clinical parameters. AFF3 gene encodes a tissue-restricted nuclear transcriptional activator that is preferentially expressed in lymphoid tissue. Interestingly, recent studies showed an association of AFF3 locus with triglyceride levels [45] and the end-stage renal disease [46] as a major complication of diabetes. These findings suggest even more plausible clinical implications of AFF3 for RA since serum lipid alterations are monitored and managed in RA patients to minimize the long-term risk of cardiovascular disease and diabetes [47–49]. The association of AFF3 locus with RA was first identified by Barton and co-authors [50]. Although the association of SNP in *AFF3* gene [rs11676922] was previously found in meta-analysis [30] we did not confirm it in Slovak cohort. This might be influenced by stronger association of AFF3 SNP in ACPA positive patients [40] and therefore undetected in our mixed cohort with respect to its size (as mentioned above). Several other studies reporting an association of *AFF3* gene with RA analysed different SNPs in this gene [50, 51]. In RDA analysis performed in our study, all factors entered the analysis equivalently as explanatory variables and their appearance in final model after forward selection indicates their own significant contribution to data variability, i.e. strong impact on parameters of clinical manifestation. Thus, our results not only confirm the central role of ACPA as a determinant of RA severity and or inflammatory status, but suggest significant contribution of risk variants in *AFF3* gene. Our results support the view that even if some genes, analysed as independent factors, may not provide sufficient risk stratification, their involvement within a prediction/explanatory model may help to identify clinically relevant high- and low-risk groups [15].

In this study, we confirmed an association of *PTPN22*, *STAT4*, *IRF5* and *PADI4* with RA in Slovak population. A study of *PTPN22*, *STAT4*, *OLIG3/TNFAIP3*, *TRAF1/C5* and HLA-DRB1*04 in Slovak population was previously reported by Stark [52]. Unlike that study, in which authors used osteoarthritis patients as controls, our cohort consisted of gender- and geographically matched healthy individuals. Furthermore, seven additional SNPs with known association with RA were investigated and the first of its kind comprehensive analysis of *HLA-DRB1* allele prevalence in Slovak population was performed in our study.

In general, genome-wide association studies-established SNPs have helped identify cellular pathways relevant to RA pathogenesis, however, their modest effect limits their predictive value [53]. Several reasons exist for a failure to replicate GWAS findings. First, it could be that this study had insufficient power to detect the modest effect sizes of selected SNPs. Second, for some non-replicated SNPs, there are differences in terms of the allele frequencies and LD patterns. Third, the magnitude of the effect of a risk allele might be altered in combination with other genetic and non-genetic factors [54, 55]. We performed the present study on the genetically homogeneous Slovak population, which shares a common genetic and cultural background and a common environment and is characterized by good genealogical and clinical records and low migration rates, thus contributing to an increased reliability of the data collected. Interestingly, an analysis of individual SNPs, in which association with RA was confirmed such as in *PADI4*, *PTPN22*, *STAT4* or *IRF5*, were also found in the multiparametric LDA model suggesting that these genes represent independent risk factors for RA.

## Conclusion

This study shows replication data of previously identified genetic risk factors of RA in Slovak population. In single as well as multiparametric analyses, *HLA-DRB1*, *PTPN22*, *STAT4*, *IRF5* and *PADI4* significantly discriminated between RA patients and healthy controls. *IRF5*, *CD28* and *CTLA4* were associated with seropositivity in RA patients. Moreover, risk alleles in *AFF3* gene together with the presence of ACPA, were associated with higher clinical severity of RA. The association of multiple risk variants related to T cell receptor signalling with seropositivity may suggest an important role in distinct clinical phenotypes of RA. Our study demonstrates that multiparametric analyses represent a powerful tool for investigation of mutual relationships of potential risk factors in complex diseases such as RA.

## Abbreviations

RA: rheumatoid arthritis
RF: rheumatoid factor
ACPA: anti–citrullinated protein antibody
HLA: human leukocyte antigen
SE: shared epitope
CRP: C-reactive protein
DAS28: disease activity score 28
PrGA: provider global assessment of disease activity
PTPN22: protein tyrosine phosphatase, non-receptor type 22 (lymphoid]
STAT4: signal transducer and activator of transcription 4
CTLA4: cytotoxic T-lymphocyte-associated protein 4
TRAF1/C5: tumour necrosis factor-receptor associated factor 1/complement component 5
CSK: C-terminal Src kinase
CD28: cluster of differentiation 28
AFF3: AF4/FMR2 family member 3
IRF5: interferon regulatory factor 5
TNFAIP3: tumour necrosis factor, alpha-induced protein 3
PADI4: peptidyl arginine deiminase, type IV
LDA: linear discriminant analysis
RDA: redundancy analysis
SNP: single nucleotide polymorphism

## References

[1] van der Helm-van Mil AH, Toes RE, Huizinga TW. Genetic variants in the prediction of rheumatoid arthritis. Ann Rheum Dis. 2010;69(9]:1694–6. 10.1136/ard.2009.123828 20439292

[2] Stastny P. Mixed lymphocyte cultures in rheumatoid arthritis. J Clin Invest. 1976;57(5]:1148–57. 1262462 10.1172/JCI108382 PMC436767

[3] Okada Y, Wu D, Trynka G, Raj T, Terao C, Ikari K, et al Genetics of rheumatoid arthritis contributes to biology and drug discovery. Nature. 2014;506(7488]:376–81. 10.1038/nature12873 24390342 PMC3944098

[4] Raychaudhuri S, Sandor C, Stahl EA, Freudenberg J, Lee HS, Jia X, et al Five amino acids in three HLA proteins explain most of the association between MHC and seropositive rheumatoid arthritis. Nat Genet. 2012;44(3]:291–6. 10.1038/ng.1076 22286218 PMC3288335

[5] Ding B, Padyukov L, Lundstrom E, Seielstad M, Plenge RM, Oksenberg JR, et al Different patterns of associations with anti-citrullinated protein antibody-positive and anti-citrullinated protein antibody-negative rheumatoid arthritis in the extended major histocompatibility complex region. Arthritis Rheum. 2009;60(1]:30–8. 10.1002/art.24135 19116921 PMC2874319

[6] Huizinga TW, Amos CI, van der Helm-van Mil AH, Chen W, van Gaalen FA, Jawaheer D, et al Refining the complex rheumatoid arthritis phenotype based on specificity of the HLA-DRB1 shared epitope for antibodies to citrullinated proteins. Arthritis Rheum. 2005;52(11]:3433–8. 16255021 10.1002/art.21385

[7] Viatte S, Massey J, Bowes J, Duffus K, arc OC, Eyre S, et al Replication of genetic loci outside the HLA conferring susceptibility to anti-CCP negative rheumatoid arthritis. Arthritis & rheumatology. 2016. 10.1002/art.39619 PMC4924598 26895230

[8] Padyukov L, Seielstad M, Ong RT, Ding B, Ronnelid J, Seddighzadeh M, et al A genome-wide association study suggests contrasting associations in ACPA-positive versus ACPA-negative rheumatoid arthritis. Ann Rheum Dis. 2011;70(2]:259–65. 10.1136/ard.2009.126821 21156761 PMC3015094

[9] Eyre S, Bowes J, Diogo D, Lee A, Barton A, Martin P, et al High-density genetic mapping identifies new susceptibility loci for rheumatoid arthritis. Nat Genet. 2012;44(12]:1336–40. 10.1038/ng.2462 23143596 PMC3605761

[10] Liu C, Ackerman HH, Carulli JP. A genome-wide screen of gene-gene interactions for rheumatoid arthritis susceptibility. Human genetics. 2011;129(5]:473–85. 10.1007/s00439-010-0943-z 21210282

[11] McClure A, Lunt M, Eyre S, Ke X, Thomson W, Hinks A, et al Investigating the viability of genetic screening/testing for RA susceptibility using combinations of five confirmed risk loci. Rheumatology (Oxford]. 2009;48(11]:1369–74. 10.1093/rheumatology/kep272 PMC2762544 19741008

[12] Karlson EW, Chibnik LB, Kraft P, Cui J, Keenan BT, Ding B, et al Cumulative association of 22 genetic variants with seropositive rheumatoid arthritis risk. Ann Rheum Dis. 2010;69(6]:1077–85. 10.1136/ard.2009.120170 20233754 PMC2933175

[13] Chibnik LB, Keenan BT, Cui J, Liao KP, Costenbader KH, Plenge RM, et al Genetic risk score predicting risk of rheumatoid arthritis phenotypes and age of symptom onset. PLoS One. 2011;6(9]:e24380 10.1371/journal.pone.0024380 21931699 PMC3171415

[14] Karlson EW, Ding B, Keenan BT, Liao K, Costenbader KH, Klareskog L, et al Association of environmental and genetic factors and gene-environment interactions with risk of developing rheumatoid arthritis. Arthritis care & research. 2013;65(7]:1147–56. 23495093 10.1002/acr.22005 PMC3740546

[15] Scott IC, Seegobin SD, Steer S, Tan R, Forabosco P, Hinks A, et al Predicting the risk of rheumatoid arthritis and its age of onset through modelling genetic risk variants with smoking. PLoS Genet. 2013;9(9]:e1003808 10.1371/journal.pgen.1003808 24068971 PMC3778023

[16] Janssens AC, Pardo MC, Steyerberg EW, van Duijn CM. Revisiting the clinical validity of multiplex genetic testing in complex diseases. Am J Hum Genet. 2004;74(3]:585–8; author reply 8–9. 14973786 10.1086/382052 PMC1182273

[17] Meigs JB, Shrader P, Sullivan LM, McAteer JB, Fox CS, Dupuis J, et al Genotype score in addition to common risk factors for prediction of type 2 diabetes. N Engl J Med. 2008;359(21]:2208–19. 10.1056/NEJMoa0804742 19020323 PMC2746946

[18] Lu Q, Song Y, Wang X, Won S, Cui Y, Elston RC. The effect of multiple genetic variants in predicting the risk of type 2 diabetes. BMC proceedings. 2009;3 Suppl 7:S49 20018041 10.1186/1753-6561-3-s7-s49 PMC2795948

[19] Patterson N, Price AL, Reich D. Population structure and eigenanalysis. PLoS Genet. 2006;2(12]:e190 17194218 10.1371/journal.pgen.0020190 PMC1713260

[20] Price AL, Patterson NJ, Plenge RM, Weinblatt ME, Shadick NA, Reich D. Principal components analysis corrects for stratification in genome-wide association studies. Nat Genet. 2006;38(8]:904–9. 16862161 10.1038/ng1847

[21] Aletaha D, Neogi T, Silman AJ, Funovits J, Felson DT, Bingham CO 3rd, et al 2010 Rheumatoid arthritis classification criteria: an American College of Rheumatology/European League Against Rheumatism collaborative initiative. Arthritis Rheum. 2010;62(9]:2569–81. 10.1002/art.27584 20872595

[22] Fransen J, van Riel PL. The Disease Activity Score and the EULAR response criteria. Clin Exp Rheumatol. 2005;23(5 Suppl 39]:S93–9. 16273792

[23] Fries JF, Spitz P, Kraines RG, Holman HR. Measurement of patient outcome in arthritis. Arthritis Rheum. 1980;23(2]:137–45. 7362664 10.1002/art.1780230202

[24] Holoshitz J. The rheumatoid arthritis HLA-DRB1 shared epitope. Curr Opin Rheumatol. 2010;22(3]:293–8. 10.1097/BOR.0b013e328336ba63 20061955 PMC2921962

[25] Iwamoto T, Ikari K, Nakamura T, Kuwahara M, Toyama Y, Tomatsu T, et al Association between PADI4 and rheumatoid arthritis: a meta-analysis. Rheumatology (Oxford]. 2006;45(7]:804–7. 10.1093/rheumatology/kel023 16449362

[26] Martinez A, Valdivia A, Pascual-Salcedo D, Lamas JR, Fernandez-Arquero M, Balsa A, et al PADI4 polymorphisms are not associated with rheumatoid arthritis in the Spanish population. Rheumatology (Oxford]. 2005;44(10]:1263–6. 10.1093/rheumatology/kei008 15998632

[27] Burr ML, Naseem H, Hinks A, Eyre S, Gibbons LJ, Bowes J, et al PADI4 genotype is not associated with rheumatoid arthritis in a large UK Caucasian population. Ann Rheum Dis. 2010;69(4]:666–70. 10.1136/ard.2009.111294 19470526 PMC2927647

[28] Dieguez-Gonzalez R, Calaza M, Perez-Pampin E, de la Serna AR, Fernandez-Gutierrez B, Castaneda S, et al Association of interferon regulatory factor 5 haplotypes, similar to that found in systemic lupus erythematosus, in a large subgroup of patients with rheumatoid arthritis. Arthritis Rheum. 2008;58(5]:1264–74. 10.1002/art.23426 18438842

[29] Wang C, Kokkonen H, Sandling JK, Johansson M, Seddighzadeh M, Padyukov L, et al Preferential association of interferon regulatory factor 5 gene variants with seronegative rheumatoid arthritis in 2 Swedish case-control studies. J Rheumatol. 2011;38(10]:2130–2. 10.3899/jrheum.110322 21807777

[30] Stahl EA, Raychaudhuri S, Remmers EF, Xie G, Eyre S, Thomson BP, et al Genome-wide association study meta-analysis identifies seven new rheumatoid arthritis risk loci. Nat Genet. 2010;42(6]:508–14. 10.1038/ng.582 20453842 PMC4243840

[31] Kurreeman F, Liao K, Chibnik L, Hickey B, Stahl E, Gainer V, et al Genetic basis of autoantibody positive and negative rheumatoid arthritis risk in a multi-ethnic cohort derived from electronic health records. Am J Hum Genet. 2011;88(1]:57–69. 10.1016/j.ajhg.2010.12.007 21211616 PMC3014362

[32] van der Helm-van Mil AH, Verpoort KN, Breedveld FC, Huizinga TW, Toes RE, de Vries RR. The HLA-DRB1 shared epitope alleles are primarily a risk factor for anti-cyclic citrullinated peptide antibodies and are not an independent risk factor for development of rheumatoid arthritis. Arthritis Rheum. 2006;54(4]:1117–21. 16572446 10.1002/art.21739

[33] Karlson EW, Chibnik LB, Cui J, Plenge RM, Glass RJ, Maher NE, et al Associations between human leukocyte antigen, PTPN22, CTLA4 genotypes and rheumatoid arthritis phenotypes of autoantibody status, age at diagnosis and erosions in a large cohort study. Ann Rheum Dis. 2008;67(3]:358–63. 17666451 10.1136/ard.2007.071662 PMC2945890

[34] Chang Foreman HC, Van Scoy S, Cheng TF, Reich NC. Activation of interferon regulatory factor 5 by site specific phosphorylation. PLoS One. 2012;7(3]:e33098 10.1371/journal.pone.0033098 22412986 PMC3297630

[35] Romo-Tena J, Gomez-Martin D, Alcocer-Varela J. CTLA-4 and autoimmunity: new insights into the dual regulator of tolerance. Autoimmun Rev. 2013;12(12]:1171–6. 10.1016/j.autrev.2013.07.002 23851140

[36] Han TU, Bang SY, Kang C, Bae SC. TRAF1 polymorphisms associated with rheumatoid arthritis susceptibility in Asians and in Caucasians. Arthritis Rheum. 2009;60(9]:2577–84. 10.1002/art.24759 19714643

[37] Gough SC, Walker LS, Sansom DM. CTLA4 gene polymorphism and autoimmunity. Immunol Rev. 2005;204:102–15. 15790353 10.1111/j.0105-2896.2005.00249.x

[38] Plenge RM, Padyukov L, Remmers EF, Purcell S, Lee AT, Karlson EW, et al Replication of putative candidate-gene associations with rheumatoid arthritis in >4,000 samples from North America and Sweden: association of susceptibility with PTPN22, CTLA4, and PADI4. Am J Hum Genet. 2005;77(6]:1044–60. 16380915 10.1086/498651 PMC1285162

[39] Costenbader KH, Chang SC, De Vivo I, Plenge R, Karlson EW. Genetic polymorphisms in PTPN22, PADI-4, and CTLA-4 and risk for rheumatoid arthritis in two longitudinal cohort studies: evidence of gene-environment interactions with heavy cigarette smoking. Arthritis Res Ther. 2008;10(3]:R52 10.1186/ar2421 18462498 PMC2483441

[40] Viatte S, Plant D, Bowes J, Lunt M, Eyre S, Barton A, et al Genetic markers of rheumatoid arthritis susceptibility in anti-citrullinated peptide antibody negative patients. Ann Rheum Dis. 2012;71(12]:1984–90. 10.1136/annrheumdis-2011-201225 22661644 PMC3595982

[41] De Rycke L, Peene I, Hoffman IE, Kruithof E, Union A, Meheus L, et al Rheumatoid factor and anticitrullinated protein antibodies in rheumatoid arthritis: diagnostic value, associations with radiological progression rate, and extra-articular manifestations. Ann Rheum Dis. 2004;63(12]:1587–93. 15547083 10.1136/ard.2003.017574 PMC1754859

[42] Meyer O, Nicaise-Roland P, Santos MD, Labarre C, Dougados M, Goupille P, et al Serial determination of cyclic citrullinated peptide autoantibodies predicted five-year radiological outcomes in a prospective cohort of patients with early rheumatoid arthritis. Arthritis Res Ther. 2006;8(2]:R40 16469118 10.1186/ar1896 PMC1526612

[43] van der Helm-van Mil AH, Verpoort KN, Breedveld FC, Toes RE, Huizinga TW. Antibodies to citrullinated proteins and differences in clinical progression of rheumatoid arthritis. Arthritis Res Ther. 2005;7(5]:R949–58. 16207336 10.1186/ar1767 PMC1257421

[44] Knevel R, de Rooy DP, Zhernakova A, Grondal G, Krabben A, Steinsson K, et al Association of variants in IL2RA with progression of joint destruction in rheumatoid arthritis. Arthritis Rheum. 2013;65(7]:1684–93. 10.1002/art.37938 23529819

[45] Li C, Bazzano LA, Rao DC, Hixson JE, He J, Gu D, et al Genome-wide linkage and positional association analyses identify associations of novel AFF3 and NTM genes with triglycerides: the GenSalt study. Journal of genetics and genomics = Yi chuan xue bao. 2015;42(3]:107–17. 10.1016/j.jgg.2015.02.003 25819087 PMC4761343

[46] Sandholm N, Salem RM, McKnight AJ, Brennan EP, Forsblom C, Isakova T, et al New susceptibility loci associated with kidney disease in type 1 diabetes. PLoS Genet. 2012;8(9]:e1002921 10.1371/journal.pgen.1002921 23028342 PMC3447939

[47] Kerekes G, Nurmohamed MT, Gonzalez-Gay MA, Seres I, Paragh G, Kardos Z, et al Rheumatoid arthritis and metabolic syndrome. Nat Rev Rheumatol. 2014;10(11]:691–6. 10.1038/nrrheum.2014.121 25090948

[48] Steiner G, Urowitz MB. Lipid profiles in patients with rheumatoid arthritis: mechanisms and the impact of treatment. Semin Arthritis Rheum. 2009;38(5]:372–81. 10.1016/j.semarthrit.2008.01.015 18395771

[49] Ursini F, Russo E, D'Angelo S, Arturi F, Hribal ML, D'Antona L, et al Prevalence of Undiagnosed Diabetes in Rheumatoid Arthritis: an OGTT Study. Medicine. 2016;95(7]:e2552 10.1097/MD.0000000000002552 26886599 PMC4998599

[50] Barton A, Eyre S, Ke X, Hinks A, Bowes J, Flynn E, et al Identification of AF4/FMR2 family, member 3 (AFF3] as a novel rheumatoid arthritis susceptibility locus and confirmation of two further pan-autoimmune susceptibility genes. Hum Mol Genet. 2009;18(13]:2518–22. 10.1093/hmg/ddp177 19359276 PMC2694689

[51] Plant D, Flynn E, Mbarek H, Dieude P, Cornelis F, Arlestig L, et al Investigation of potential non-HLA rheumatoid arthritis susceptibility loci in a European cohort increases the evidence for nine markers. Ann Rheum Dis. 2010;69(8]:1548–53. 10.1136/ard.2009.121020 20498205 PMC2938898

[52] Stark K, Rovensky J, Blazickova S, Grosse-Wilde H, Ferencik S, Hengstenberg C, et al Association of common polymorphisms in known susceptibility genes with rheumatoid arthritis in a Slovak population using osteoarthritis patients as controls. Arthritis Res Ther. 2009;11(3]:R70 10.1186/ar2699 19445664 PMC2714116

[53] Plenge RM. Recent progress in rheumatoid arthritis genetics: one step towards improved patient care. Curr Opin Rheumatol. 2009;21(3]:262–71. 10.1097/BOR.0b013e32832a2e2d 19365266

[54] Ioannidis JP. Non-replication and inconsistency in the genome-wide association setting. Human heredity. 2007;64(4]:203–13. 17551261 10.1159/000103512

[55] Weir BS, Cardon LR, Anderson AD, Nielsen DM, Hill WG. Measures of human population structure show heterogeneity among genomic regions. Genome research. 2005;15(11]:1468–76. 16251456 10.1101/gr.4398405 PMC1310634

